# Artificial Intelligence and Digital Pathology for Preoperative Lymphovascular Invasion and Metastatic Risk Prediction in Breast Cancer: Bridging the Biopsy–Resection Gap

**DOI:** 10.3390/jcm15145381

**Published:** 2026-07-09

**Authors:** Bandar S. Alshreef, Yousif A. Kariri

**Affiliations:** Department of Clinical Laboratory Sciences, College of Applied Medical Sciences, Shaqra University, Shaqra 11961, Saudi Arabia; bsalshreef@su.edu.sa

**Keywords:** breast cancer, lymphovascular invasion, digital pathology, artificial intelligence, magnetic resonance imaging, biopsy

## Abstract

Breast cancer is a leading global diagnosis where lymphovascular invasion (LVI) serves as a critical indicator of metastatic potential. Conventional assessment is often hindered by its reliance on postoperative findings, sampling errors, and subjective interobserver variability. This review evaluates how artificial intelligence (AI), digital pathology, and MRI radiomics provide earlier, quantitative estimations of LVI-related risk. The strongest direct evidence currently comes from MRI-based studies, where validation performance often reaches area under the receiver operating characteristic curve (AUC) values of 0.84–0.90. By contrast, digital pathology is especially mature for LVI-adjacent tasks such as lymph node metastasis detection and slide-based relapse-risk modelling, which together provide an important translational foundation for future LVI-specific tools. This review also addresses issues that remain underdeveloped in the literature, including the distinction between lymphatic and vascular invasion, AI-specific risk-of-bias and reporting frameworks, the emerging regulatory landscape for adjacent breast AI tools, and the gap between resection-based development datasets and biopsy-level preoperative use. Although most modern computational models are developed using extensive surgical resection specimens, their true clinical utility hinges on successful validation and performance within the highly restricted, fragmented tissue context of preoperative core needle biopsies. Overall, the field appears most promising when LVI prediction is framed not as an isolated binary task, but as one component of a broader metastatic-risk workflow that supports calibrated, multidisciplinary breast cancer decision-making.

## 1. Introduction

Breast cancer has become the most frequently diagnosed cancer in women worldwide, and disease progression remains closely tied to regional and distant metastatic spread [1]. In everyday breast oncology practice, decisions about axillary staging, systemic therapy, and surveillance all depend on how confidently clinicians can estimate metastatic potential before and after surgery. This is precisely where lymphovascular invasion (LVI) has enduring clinical importance. Histologically, LVI denotes tumor emboli within endothelial-lined lymphatic or vascular channels and has repeatedly been associated with nodal involvement, recurrence, and poorer survival [2].

Yet the current use of LVI in practice is narrower than its biologic relevance [3]. In many centers it is treated as a postoperative binary variable rather than as a rich morphologic phenotype, and its detection can be confounded by retraction artifact, sampling limitations, and differences in pathologist interpretation. As a result, a marker that reflects metastatic capability is often recognized late and expressed in a simplified format that does not fully capture tumor behavior [2].

In parallel, breast pathology has undergone a digital transformation. Whole-slide imaging has enabled computational pathology workflows in which algorithms can evaluate tissue architecture, tumor–stroma interactions, immune infiltrates, biomarker expression, and lymph node metastases at scales not feasible in routine visual review [4,5,6,7]. This evolution creates a natural bridge between conventional LVI assessment and a broader artificial intelligence (AI)-enabled framework for predicting metastatic risk from both pathology and imaging data.

**Figure 1:**
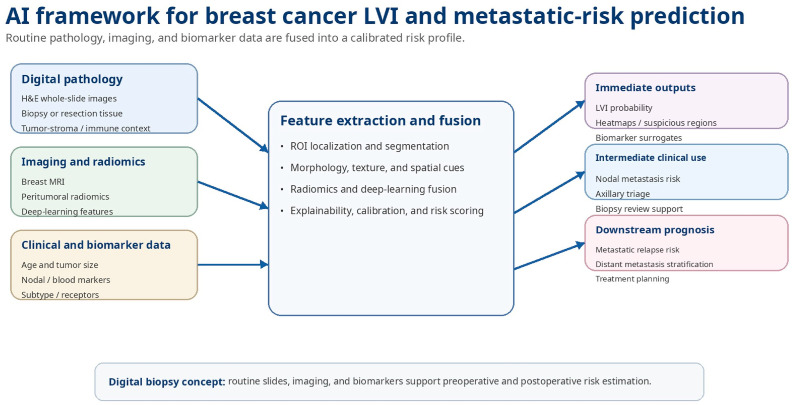
**Conceptual framework for AI-enabled prediction of lymphovascular invasion and metastatic risk in breast cancer.** The figure shows how digitized pathology, magnetic resonance imaging (MRI)/radiomic features, clinical variables, and biomarker data can be integrated to estimate LVI probability, nodal involvement, and downstream metastatic risk.

This review addresses these challenges by presenting a distinct, critical viewpoint: interpreting LVI as an isolated, postoperative binary checkbox fails to capture its rich biological phenotype and restricts its preoperative clinical utility. We propose that the true translational potential of AI lies not in developing isolated LVI classifiers, but in establishing a hierarchical, multimodal ‘digital biopsy’ framework. Rather than predicting LVI as an endpoint, this framework conceptualizes LVI as a continuous phenotypic surrogate of regional and systemic disease dissemination. By synthesizing evidence across digital pathology, Magnetic Resonance Imaging (MRI) radiomics, and clinical biomarkers, this approach transforms routine, fragmented diagnostic data into a unified, calibrated metastatic-risk ecosystem. Ultimately, we provide a definitive roadmap arguing that AI-enabled LVI prediction must be operationalized as a continuous phenotypic estimator of regional and systemic spread, shifting the clinical workflow from retrospective documentation to proactive, multidisciplinary decision-support. This conceptual framework, linking multi-source data fusion to immediate outputs and downstream risk stratification, is structurally summarized in Figure 1.

### Literature Selection

This manuscript was prepared as a narrative review. Relevant publications were identified through searches of PubMed, Scopus, and Web of Science using combinations of the terms breast cancer, LVI, AI, digital pathology, whole-slide imaging, radiomics, deep learning, magnetic resonance imaging, and metastatic risk prediction. Priority was given to English-language articles published between 2019 and 2025, while earlier landmark studies were included where necessary to provide historical and scientific context. Studies were selected according to their methodological quality, clinical relevance, originality, and contribution to AI-based approaches for LVI assessment and metastatic risk prediction. Rather than aiming for an exhaustive systematic evaluation of all published studies, this review synthesizes representative evidence to critically examine current advances, identify knowledge gaps, and discuss future research priorities.

## 2. Clinical Significance of LVI and Limits of Conventional Assessment

LVI is not a trivial ancillary finding. Meta-analytic evidence indicates that the presence of LVI is associated with worse disease-free and overall survival in breast cancer, reinforcing its value as an adverse prognostic factor rather than a mere descriptive observation [4]. Clinically, LVI has also been linked to non-sentinel nodal disease, greater locoregional burden, and more aggressive tumor biology.

However, routine assessment remains constrained in at least four ways. First, LVI is often known only after definitive surgery, limiting its usefulness for preoperative triage. Second, recognition on hematoxylin-eosin sections can be difficult when artefactual spaces mimic vascular channels. Third, standard pathology reporting usually reduces a complex morphologic process to present-versus-absent language. Fourth, the conventional workflow rarely integrates LVI with surrounding stromal, immune, and radiologic context in a quantitative manner. These shortcomings explain why AI-based approaches are attractive: they promise not merely automation, but richer measurement.

## 3. Distinguishing Lymphatic from Vascular Invasion

A further conceptual refinement is important here. Although routine pathology reports often use the composite term LVI, lymphatic invasion and blood-vessel invasion are not biologically identical. Studies that have separated these processes suggest that their prognostic weight may differ across molecular subtypes, while contemporary reviews emphasize that standard hematoxylin-eosin assessment does not reliably distinguish them in routine practice. For AI development, this matters because training labels that collapse lymphatic and vascular invasion into a single endpoint may obscure biologically meaningful signal. Researchers can overcome the limitations of traditional binary reporting for LVI by implementing multi-task learning (MTL) architectures. Instead of integrating lymphatic and blood vascular invasion into a larger “LVI” category, MTL allows a unified model to extract common diagnostic characteristics while using separate output channels for each kind of invasion. This methodology maintains the high-resolution phenotypic features essential to investigate the distinct prognostic impact of each invasive route across several genetic subtypes of breast cancer. Future datasets should therefore retain separate annotations whenever immunohistochemistry or expert review permits, even if the primary clinical model still reports a composite LVI probability [3,8,9,10]. Remarkably, when an AI model is designed to distinguish lymphatic invasion from vascular invasion as a discrete clinical outcome, its development and validation should rely on robust reference standards, such as immunohistochemical confirmation (e.g., D2-40 for lymphatic endothelium and CD31 for vascular endothelium) or consensus annotations from multiple expert pathologists [11]. In contrast, models trained solely on routine H&E-stained slides without independent validation are unlikely to achieve reliable sub-classification because of the intrinsic limitations of tissue morphology and staining artifacts.

## 4. Digital Pathology as the Foundation for LVI-Aware Risk Modelling

Breast computational pathology has advanced rapidly over the last five years. Reviews published in 2023–2025 describe a mature ecosystem of AI applications in diagnosis, grading, biomarker quantification, tumor microenvironment analysis, lymph node metastasis detection, and outcome prediction [4,5,6,7]. The key message across these reviews is consistent: once tissue is digitized, pathologic interpretation can be supplemented by scalable quantitative analysis.

What makes this especially relevant to LVI is that LVI rarely exists in isolation. It emerges in a microenvironment shaped by tumor architecture, stromal remodeling, angiogenesis, immune composition, and invasive fronts. Digital pathology is well positioned to quantify such contextual features. In practical terms, even when slide-level algorithms are not yet explicitly trained to classify breast LVI at scale, they can still extract features that are biologically adjacent to LVI and relevant to metastatic behavior. Accordingly, the most realistic near-term vision is not an all-or-nothing ‘LVI detector’, but a layered pathology model that combines direct morphologic cues, surrounding tissue context, and downstream prognostic outputs. That framing better matches both the current evidence base and the way computational pathology is progressing in breast oncology.

A major technical hurdle in this area is the transition between different sample types. Although whole-slide images (WSIs) of surgical resections provide extensive spatial detail across an entire tumor block, preoperative core needle biopsies yield much smaller, highly restricted tissue samples. Consequently, AI models designed for surgical slides frequently struggle with biopsies due to issues like tissue fragmentation, crush artifacts, and severe undersampling of the tumor-host interface where LVI is most active. To successfully shift digital pathology from a retrospective automation tool to a proactive preoperative risk predictor, models must be intentionally trained to identify subtle, localized micro-features within these limited biopsy samples.

## 5. Histology-Based AI and Hierarchical Metastatic-Risk Modelling

Direct breast-specific histology models for LVI are still relatively limited but demonstrate a clear shift from treating LVI as a binary marker to a measurable morphologic phenotype. For instance, Chen and colleagues used supervised deep learning to quantify lymph-blood vessel invasion (LBVI) morphology in invasive ductal carcinoma, demonstrating that detailed morphometric descriptors carry critical prognostic information regarding lymph node metastasis beyond routine binary reporting [12]. The feasibility of algorithm-assisted workflows is further supported by proof-of-concept work in other malignancies, such as testicular germ cell tumors, where deep-learning models successfully highlighted regions suspicious for LVI to serve as effective clinician-facing decision-support tools [13].

Rather than viewing LVI prediction as an isolated classification task, the evolving literature highlights the clinical value of hierarchical risk modelling. In this framework, estimating microscopic invasive behavior serves as an upstream entry point to predicting regional nodal spread and systemic outcomes. To ensure methodological clarity, these biologically related but methodologically distinct endpoints are evaluated below by their respective clinical targets and input modalities.

### 5.1. Histology-Based Quantification on Resection Slides vs. Preoperative Prediction

Although specialized breast histology models for evaluating LVI are beginning to emerge, it is important to distinguish retrospective morphologic quantification performed on surgical resection specimens from true preoperative prediction using core needle biopsy (CNB) samples. Current breast pathology studies, including that of Chen et al., were developed and validated on extensive resection whole-slide images, where complete tumor architecture and vascular interfaces are available [12]. These models therefore demonstrate the feasibility of AI-assisted morphologic quantification of LBVI rather than preoperative biopsy-based prediction. In contrast, true preoperative prediction from CNB specimens remains largely unexplored and represents a key translational priority. Developing such models will require algorithms that can accommodate the limited tissue volume, fragmentation, sampling bias, and absence of the full tumor-host interface characteristic of biopsy specimens. Consequently, evidence generated from surgical resection slides should be viewed as an important foundation rather than direct evidence of clinically deployable preoperative prediction.

### 5.2. Regional Nodal Spread

At the regional level, the methodology shifts from primary tumor channel invasion to nodal burden assessment. Challa and colleagues demonstrated that AI-assisted review of lymph node slides achieved exceptional sensitivity and negative predictive value for metastasis detection while significantly reducing pathologist review times [14]. Similarly, LVI-focused magnetic resonance imaging (MRI) models increasingly integrate regional axillary signs and peritumoral edema to improve preoperative staging [15].

### 5.3. Distant Metastasis and Relapse

On the systemic level, the endpoints expand to long-term survival projections and distant recurrence.

- Digital Pathology Modality: On the primary-tumor level, Garberis and colleagues validated a deep-learning model capable of predicting 5-year metastasis-free survival directly from digitized histology slides in estrogen receptor-positive, HER2-negative early breast cancer [16].
- Multimodal and Clinicomics Pipelines: To strengthen these predictions, multimodal and clinicomics pipelines are utilized to link localized features with systemic markers. Zhang and colleagues established a clinicomics-guided distant metastasis prediction model fusing clinical and imaging variables, outperforming traditional single-modality nomograms [17].
- Low-Cost Alternatives: Moving toward low-cost, highly accessible alternatives, Tan and colleagues utilized LightGBM models to integrate clinical blood markers with ultrasound max diameter for robust distant metastasis discrimination [18]. In a parallel dual-centre cohort study, clinical blood biomarkers alone were leveraged via machine learning to accurately triage the risk of de novo distant bone metastasis [19].

**Figure 2:**
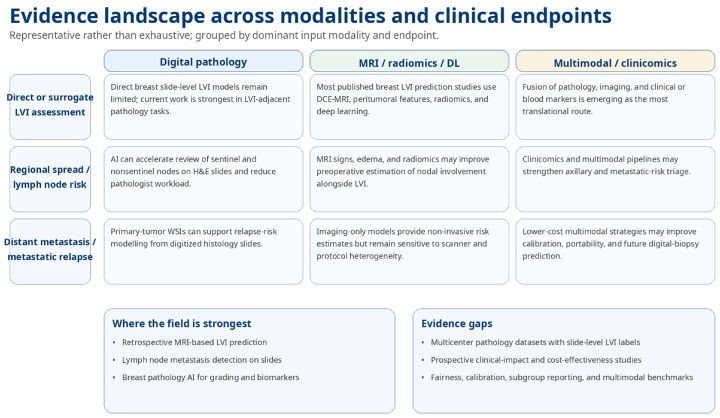
**Evidence landscape across modalities and clinical endpoints.** The figure situates direct LVI prediction within related tasks, including pathology-based LVI or LBVI quantification, lymph node metastasis detection, MRI/radiomics models, biomarker-based metastasis prediction, and slide-based relapse-risk estimation.

Ultimately, transitioning from a standalone “LVI detector” to an integrated risk engine aligns computational workflows directly with multidisciplinary cancer decision-making. By linking immediate morphological outputs to intermediate nodal status and downstream long-term survival projections, these systems provide a holistic, calibrated metastatic-risk profile. This cross-modal evidence landscape is visually summarized in Figure 2.

## 6. MRI, Radiomics, and Deep Learning for Preoperative LVI Prediction

The most substantial literature directly targeting breast LVI prediction currently comes from MRI-based radiomics and deep-learning studies. Early work showed that dynamic contrast-enhanced MRI could capture radiomic patterns associated with LVI before surgery [20]. Subsequent studies confirmed that this signal was reproducible enough to support machine-learning and nomogram-based workflows [21,22].

Importantly, recent models have moved beyond simple intratumoral analysis. They increasingly incorporate peritumoral edema, axillary-node features, delayed phases of enhancement, and deep-learning representations of both intratumoral and peritumoral regions [15,22,23,24,25,26]. This trend reflects an important biologic insight: LVI is not just a property of the tumor core, but of the tumor-host interface and surrounding microenvironment.

Representative 2024 studies illustrate how the field is evolving. In node-negative invasive breast cancer, Liang and colleagues reported strong validation performance for an MRI-based deep-learning model, with the best MLP-radiomic configuration reaching an area under the receiver operating characteristic curve (AUC) of 0.896 and outperforming a clinical random-forest baseline [23]. Liu and colleagues showed that a hybrid model combining multimodal MRI findings, radiomics, and deep-learning features achieved an AUC of 0.872, again supporting the value of integrated imaging representations [24]. Other studies in 2024 using joint MRI morphological features, radiomics, and deep learning, or dynamic and multiphase DCE-MRI nomograms, similarly found that combined models outperformed single-input approaches [15,22,24,25].

The broader pattern is reinforced by meta-analytic evidence. Ma and colleagues found that preoperative MRI-based radiomics performs well overall for predicting LVI in breast cancer, although the literature remains heterogeneous and methodologically variable [27]. In other words, the discriminative signal is real, but the field still needs more standardization before these models can be regarded as interchangeable or immediately transportable across sites.

One limitation should be emphasized clearly. Most of these studies are retrospective, single-country and scanner-specific. Performance estimates are often strongest in internal or temporally proximate validation sets, and only a minority of studies have robust external testing. Thus, while MRI-based LVI prediction shows a real discriminative signal, there remains a substantial gap between this promising statistical discrimination and validated clinical usefulness. High area under the receiver operating characteristic curve (AUC) values do not guarantee real-world transportability or a positive impact on patient outcomes; consequently, these tools are not yet deployment-ready in a routine clinical sense.

### Human-Expert Context and Interpretability

The practical question is not only whether an AI model performs well statistically, but how that performance compares with conventional expert feature interpretation. Earlier breast MRI work reported only moderate-to-substantial interobserver agreement for some LVI-related imaging findings, illustrating the limits of purely qualitative assessment [26]. Against that background, pooled MRI-radiomics performance in meta-analysis has generally fallen in the mid-to-high 0.80 range, and recent deep-learning/radiomics hybrids have reported validation AUCs approaching 0.90 [21,23]. These comparisons do not yet replace direct prospective head-to-head trials against radiologists, but they do suggest that AI can capture preoperative information beyond what is typically recoverable from routine human scoring alone.

Table 1 summarizes the methodological characteristics, validation strategies, and reported diagnostic performance of the included AI models. Although many recent deep learning and radiomics models achieved strong discrimination (AUC 0.821–0.896), important methodological limitations remain. Reporting of clinically relevant metrics such as positive and negative predictive values (PPV and NPV) is inconsistent, and model calibration and threshold optimization are rarely described outside more recent studies. In addition, most models have been developed and tested using single-centre datasets, with only a few undergoing independent external validation. These findings highlight the need for prospective multi-center studies, together with decision curve analysis and health-economic evaluation, before these models can be considered for routine clinical implementation.

## 7. Why Multimodal Digital Biopsy Models May Be the Most Useful Next Step

No single modality fully captures metastatic competence. Histology captures tissue architecture and invasive phenotype; MRI captures tumor extent, vascularity, and peritumoral changes; blood-based biomarkers offer scalable systemic signals; and routine clinical variables contextualize all of them. The emerging literature therefore points toward multimodal fusion as the most clinically meaningful direction [17,18,19,24].

In practical terms, a multimodal digital-biopsy model (i.e., a composite risk estimate derived from digitized pathology slides, MRI/radiomic features, and clinical or laboratory biomarkers) would combine slide-derived morphologic signals, MRI/radiomic features, and accessible clinical or laboratory variables into a composite risk profile. Such a profile could estimate at least three related probabilities: LVI positivity, nodal spread, and future metastatic relapse. This unified approach is attractive because it aligns more closely with actual multidisciplinary decision-making than a single-task classifier does.

**Figure 3:**
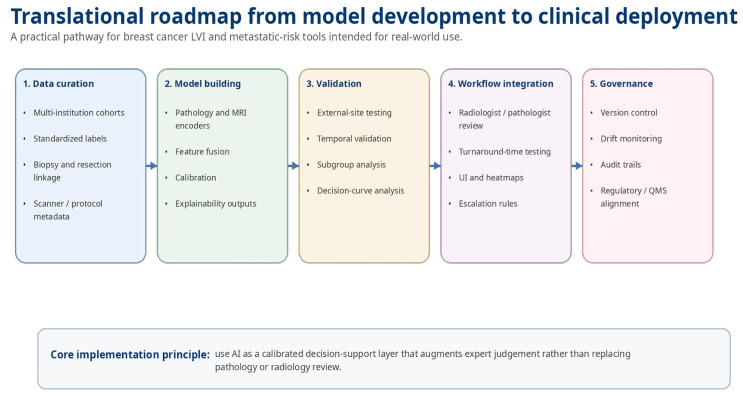
**Translational roadmap from model development to clinical deployment.** The roadmap highlights dataset assembly, expert annotation, internal validation, external testing, calibration, explainability, workflow integration, governance, and post-deployment monitoring before clinical use.

Multimodal models may also help solve an interpretability problem. A purely black-box risk score can be difficult for clinicians to trust. By contrast, a fused model can present complementary evidence layers—for example, suspicious slide regions, high-weight peritumoral MRI features, and elevated biomarker contributions—making the final prediction easier to contextualize. This is particularly important in breast cancer, where treatment choices are often preference-sensitive and based on a bundle of risk indicators rather than on one threshold alone. A practical translational pathway from model development to deployment is outlined in Figure 3.

By explicitly correlating immediate outputs like LVI likelihood to intermediate clinical endpoints like nodal metastasis and downstream outcomes like 5-year metastasis-free survival, the model transforms from a simple “detector” to a comprehensive risk engine. This unified trajectory, shown in Figure 3, supports multidisciplinary decision-making by giving a composite risk profile based on digitized slides, MRI characteristics, and clinical indicators.

## 8. Methodological and Implementation Challenges

Because many published studies use overlapping AI frameworks while addressing different clinical objectives, distinguishing their intended applications can be challenging. To improve clarity, Table 2 summarizes the intended clinical use of each approach by outlining the analytical modality, biological input, clinical application, and prediction endpoint, thereby providing a structured overview of their respective roles in breast cancer management.

Several limitations still constrain translation. The first is endpoint heterogeneity. Different studies define LVI, nodal burden, and metastasis-related outcomes in different ways, and some conflate pathologic LVI status with broader aggressiveness. Without more standardized endpoints, model comparison remains difficult [12,15,20,21,22,23,24,25].

The second is annotation burden. High-quality slide-level LVI labels require expert review, and ambiguous foci can be especially challenging to annotate consistently. This has likely contributed to the relative scarcity of large, breast-specific LVI pathology datasets compared with datasets for tumor detection or biomarker scoring [7,28].

The third is domain shift. MRI-based radiomics models are sensitive to acquisition protocols, segmentation practices, preprocessing choices, and center-specific case mix. Digital pathology models face analogous challenges related to staining variation, scanner differences, and tissue preparation. Strong internal performance therefore does not guarantee real-world transportability [6,7,27].

The fourth is clinical integration. An AI system that predicts LVI or metastatic risk must fit within actual pathology and radiology workflows. It must be calibrated, auditable, and presented in a form that clinicians can act on. For breast cancer specifically, the question is not simply whether the model predicts correctly, but whether it changes preoperative planning, reduces unnecessary procedures, or improves risk communication without introducing unsafe overcalling.

The fifth is governance. Version control, drift monitoring, subgroup performance reporting, and quality-management alignment will be crucial if these tools are to be used in practice. For LVI-related models, governance may be especially important because the downstream clinical consequences of false positives and false negatives are asymmetric and context-dependent.

A related gap is health economics. The current literature rarely tests whether earlier AI-enabled estimation of LVI or metastatic risk would reduce avoidable procedures, shorten time to adjuvant planning, or improve use of targeted follow-up imaging. Future prospective studies should therefore examine not only diagnostic performance, but also whether these tools lower unnecessary interventions or improve workflow efficiency in ways that support reimbursement and routine adoption. While a large portion of existing models rely on surgical resections, the most fundamental translational aim and urgent priority for the field is to validate and recalibrate these systems using preoperative core needle biopsies. This shift is crucial because biopsies provide unique algorithmic challenges, such as limited tissue availability, the absence of a well-defined peripheral tumor boundary, and probable sampling errors that can lead to false-negative morphologic assessments. To further strengthen methodological rigor, future breast cancer AI studies should adhere to established reporting and quality assessment frameworks, including CLAIM 2024 and QUADAS-AI, to improve transparency, reduce bias, and enhance the reliability and reproducibility of study findings.

## 9. Structured Critical Appraisal of Risk of Bias and Reporting Quality

While the integration of digital pathology, radiomics, and clinical multi-omics yields highly promising prognostic indicators, a persistent barrier to true clinical translation is methodological opacity and heterogeneous study design. Rather than utilizing risk-of-bias and validation protocols as purely abstract theoretical benchmarks, representative examples from the compiled literature can be contextualized directly against established reporting frameworks [29]. This narrative mapping highlights how selected breast cancer models align with the core risk-of-bias domains codified by QUADAS-AI and the reporting standards demanded by the 2024 CLAIM update, focusing on data curation, model transparency, reference standard integrity, and clinical portability [30,31]. The resulting illustrative overview is detailed in Table 3.

### 9.1. Domain-by-Domain Synthesis and Methodological Recommendations

Applying these structured domains to the compiled evidence reveals that while raw discriminative capabilities are frequently high (with several multimodal validation AUCs exceeding 0.85), the structural design of these pipelines remains problematic for real-world adoption.

Under the Data Curation and Patient Selection domain, the major vulnerability stems from selection and spectrum bias. Because models are developed retrospectively on pre-curated collections of high-quality surgical resection blocks, they systematically fail to account for the real-world artifacts, tissue fragmentation, and crush boundaries common to restricted preoperative core needle biopsy workflows.

Regarding Index Test and Reference Standard harmonization, the lack of continuous calibration data represents a critical gap. Standard binary classification reporting assumes an idealized, uniform clinical threshold that fails to reflect the nuanced, preference-sensitive landscape of breast oncology. Moving forward, clinical trials and model developers must transition away from publishing isolated diagnostic performance metrics, adopting instead a reporting structure fully compliant with QUADAS-AI and CLAIM 2024 to ensure models function as safe, auditable decision-support engines within the multidisciplinary cancer ecosystem.

Regulatory precedent is also emerging, although it remains adjacent rather than LVI-specific. Current breast-related approvals and certifications are concentrated in digital pathology platforms for primary diagnosis, established image-analysis ecosystems for breast biomarkers, mitosis-counting support tools, and AI mammographic risk systems rather than dedicated breast LVI classifiers [32,33,34]. This matters because the path from research model to clinical deployment will likely require not only stronger validation, but also clearer intended-use definitions, workflow integration plans, and eventually health-economic evidence showing that these tools change decisions in a useful way.

### 9.2. Economic Viability, Reimbursement Landscapes, and Operational Integration

Clinical integration of predictive algorithms within breast cancer workflows is ultimately governed by health economic viability rather than performance metrics alone. A preoperative digital pathology model provides structural value through two main vehicles: expense reduction and workflow acceleration. Clinically, highly granular, early prognostic indicators are envisioned to inform future risk-adapted axillary planning, though such strategies remain speculative. Rather than justifying the immediate omission of sentinel node biopsies or aggressive axillary interventions, these algorithmic risk profiles must first be rigorously tested in prospective clinical-utility studies to evaluate their safety, definitive impact on surgical de-escalation, and long-term complication management costs. Structurally, computer-aided screening relieves labor bottlenecks, optimizes pathology schedules, and accelerates multi-disciplinary board planning. However, establishing standard financial reimbursement pathway models (e.g., CPT billing or NTAP allocation) demands rigorous demonstration of shifted clinical trajectories. Because existing regulatory benchmarks focus on primary digitization rather than integrated predictive risk assessment tools, prospective studies must begin logging healthcare utilization metrics to validate long-term financial efficiency and secure institutional adoption.

## 10. Future Directions

Future progress will likely depend on six priorities. First, multi-center breast datasets should be assembled with explicit slide-level or case-level LVI labels, ideally linked to MRI, nodal outcomes, and follow-up. Second, investigators should report calibration and subgroup performance more consistently, not just AUC. Third, breast biopsy slides deserve more attention, because preoperative usefulness is one of the chief reasons to pursue these models in the first place. Fourth, explainability should be operational rather than decorative, providing slide regions, imaging features, or variable contributions that clinicians can interpret. Fifth, prospective or pseudo-prospective workflow studies should test whether AI changes time, confidence, and management decisions. Sixth, multimodal benchmarks should be created so that pathology-only, imaging-only, and fused models can be compared on common tasks.

### Biopsy-Level Prediction as a Near-Term Translational Priority

Most pathology AI work relevant to LVI has been developed on surgical resections, whereas real preoperative decision-making depends on core needle biopsy material. That distinction is not trivial: breast core biopsies provide less tissue, may undersample invasive fronts, and can create interpretive challenges when evaluating features such as definite LVI [35]. Even so, recent breast AI studies have shown that pretreatment biopsy whole-slide images can support meaningful outcome prediction, including response modelling before neoadjuvant therapy [36]. A logical next step for the field is therefore the creation of matched biopsy-resection cohorts in which LVI-related morphology, nodal status, and downstream outcomes can be studied across specimen types rather than assumed to transfer automatically from one to the other.

If these steps are taken, the field could move from fragmented proof-of-concept studies to clinically meaningful decision-support systems. Such systems would not replace pathologists or radiologists. Rather, they would offer a more quantitative and integrated way to estimate occult invasion and metastatic risk using data that are already being generated in routine care.

## 11. Conclusions

The literature now supports a clear conclusion: AI has the long-term potential to contribute to the prediction of LVI and metastatic risk in breast cancer, but the contribution is currently uneven across modalities and remains restricted to experimental or retrospective validation stages. MRI-based radiomics and deep-learning workflows provide the strongest direct evidence for preoperative LVI prediction, whereas digital pathology has shown particularly strong value in adjacent tasks. However, a clear boundary must be drawn between promising discriminatory performance—which is abundantly documented via retrospective AUC metrics—and validated clinical usefulness, which has yet to be proven through prospective, multi-center trials. Before these models can transition into active clinical decision-support engines, the next phase of research must prioritize rigorous external validation, model calibration, and health-economic assessments that demonstrate an actual net clinical benefit in multi-disciplinary care settings.

## Figures and Tables

**Table 1:** Methodological Characteristics, Validation Strategies, and Diagnostic Performance of Included AI Models.

Study	Modality and Endpoint	Population and Task Level	Discrimination Performance (AUC and 95% CI)	Classification Threshold Metrics (Sens/Spec/PPV/NPV)	Validation Strategy	Model Calibration and Threshold Selection	Clinical Utility (DCA/Clinical Net Benefit)
**A. Preoperative Diagnostic LVI Prediction (Patient-Level)**							
Liu et al., 2019 [20]	DCE-MRI radiomics	Patients with invasive breast cancer undergoing preoperative DCE-MRI; patient-level preoperative prediction of histopathologic LVI	Validation AUC: 0.821 (*95% CI: NR*)	Sens: 78.6% Spec: 80.2% PPV: *NR* NPV: *NR*	Single-center exploratory cohort	Retrospective optimal cut-off threshold (Youden Index); Calibration: *NR*	No decision curve analysis (DCA) reported
Nijiati et al., 2022 [21]	MRI radiomics	Patients with invasive breast cancer undergoing preoperative multiparametric MRI; patient-level preoperative prediction of histopathologic LVI	Validation AUC: 0.843 (*95% CI: NR*)	Sens: 75.2% Spec: 83.1% PPV: *NR* NPV: *NR*	Internal training/validation split	ROC-based probability threshold; calibration curve reported	No DCA reported
Ma et al., 2023 [27]	Meta-analysis of MRI radiomics	Studies including patients with breast cancer; study-level meta-analysis of preoperative MRI for LVI prediction	Primary summary framework utilized pooled parameters instead of single aggregate AUC	Pooled Sens: 79.0% Pooled Spec: 81.0% PPV/NPV: *NR* (Heterogeneous)	Meta-analysis framework (Heterogeneous study collection)	Evaluated between-study thresholds via summary receiver operating curves (SROC)	No patient-level DCA; pooled diagnostic performance reported
Yang et al., 2024 [25]	MRI morphology + radiomics + DL	Patients with invasive breast cancer undergoing preoperative MRI; patient-level preoperative prediction of histopathologic LVI	Multimodal Hybrid AUC: 0.865 (*95% CI: NR*)	Sens: 78.0% Spec: 84.0% PPV: *NR* NPV: *NR*	Internal train/test split validation	Threshold optimized on training set; Calibration: *NR*	No DCA reported
Liang et al., 2024 [23]	MRI-based deep learning	Node-negative patients with invasive breast cancer undergoing preoperative MRI; patient-level preoperative prediction of histopathologic LVI	Validation AUC: 0.896 (*95% CI: 0.842–0.941*)	Sens: 84.3% Spec: 86.1% PPV: 79.4% NPV: 89.8%	Internal hold-out validation set	Receiver Operating Characteristic	Decision curve analysis reported
Liu et al., 2024 [24]	Multimodal MRI + radiomics + DL	Patients with invasive breast cancer undergoing preoperative MRI; patient-level preoperative prediction of histopathologic LVI	Validation AUC: 0.872 (*95% CI: 0.819–0.925*)	Sens: 81.2% Spec: 82.5% PPV: *NR* NPV: *NR*	Internal random split validation	Intratumoral/peritumoral thresholding; Hosmer–Lemeshow calibration reported	Decision curve analysis reported
Zhang et al., 2024 [26]	MRI radiomics nomogram	Patients with invasive breast cancer undergoing preoperative MRI; patient-level preoperative prediction of histopathologic LVI	Nomogram Validation AUC: 0.854 (*95% CI: NR*)	Sens: 77.5% Spec: 83.0% PPV: *NR* NPV: *NR*	Internal split validation	Fused score used for nomogram thresholding; Calibration curve was reported.	Decision curve analysis reported
**B. Histopathologic LBVI Quantification (Slide/Patch-Level)**							
Chen et al., 2023 [12]	Breast histology + DL	Patients with breast cancer; slide-level prediction of lymph-blood vessel invasion (LBVI) on histopathology	Validation AUC: 0.850 (*95% CI: NR*)	Sens: 81.0% Spec: 79.5% PPV: *NR* NPV: *NR*	Internal validation cohort	Automated morphometric threshold; Calibration NR	Continuous morphometric measurements were evaluated; no formal DCA reported
**C. Regional Lymph Node Metastasis Detection (Slide-Level Screening)**							
Challa et al., 2023 [14]	Whole-slide pathology AI	Patients with breast cancer undergoing axillary lymph node assessment; slide-level detection of lymph node metastases	Slide-level AUC: 0.962 (*95% CI: NR*)	Sens: 98.2% Spec: 89.4% PPV: 85.1% NPV: 99.1%	Single-center clinical screening workflow	Operating threshold selected for screening workflow	Operational workflow metrics reported; no formal DCA reported
**D. Distant Metastasis & Relapse Risk Modeling (Prognostic/Survival Endpoints)**							
Zhang et al., 2024 [19]	Blood biomarkers + LightGBM	Patients with newly diagnosed breast cancer; patient-level prediction of de novo distant bone metastasis	Validation AUC: 0.881 (*95% CI: NR*)	Sens: 83.3% Spec: 81.0% PPV: 76.5% NPV: 87.2%	Dual-center external validation cohort	Laboratory marker probability thresholding; Stable calibration confirmed	External validation reported; no DCA reported unless explicitly stated
Tan et al., 2024 [18]	Blood markers + ultrasound + LightGBM	Patients with breast cancer; patient-level prediction of distant metastasis using blood biomarkers and ultrasound	External Validation AUC: 0.869 (*95% CI: 0.803–0.924*)	Sens: 80.5% Spec: 83.8% PPV: 78.1% NPV: 85.9%	Independent External Validation cohort	Multi-modality clinical risk thresholding; Calibration curve evaluated	DCA was performed.
Garberis et al., 2025 [16]	Digitized histology + DL	Patients with primary breast cancer; slide-level prediction of long-term metastatic relapse risk	Validation C-Index/AUC: 0.812 (*95% CI: NR*)	Sens: 74.0% Spec: 78.5% PPV: *NR* NPV: *NR*	Internal hold-out validation	Deep-learning risk stratification score thresholds applied	Comparison with TNM staging was reported.

**Note. NR = Not Reported by the original study authors.** Abbreviations: AI, artificial intelligence; AUC, area under the receiver operating characteristic curve; CI, Confidence Interval; DCA, Decision Curve Analysis; DCE-MRI, dynamic contrast-enhanced magnetic resonance imaging; DL, deep learning; LBVI, lymph-blood vessel invasion; LVI, lymphovascular invasion; NPV, Negative Predictive Value; PPV, Positive Predictive Value; ROC, Receiver Operating Characteristic; Sens, Sensitivity; Spec, Specificity. MRI, magnetic resonance imaging.

**Table 2:** Intended-Use and Operational Unit-of-Analysis Blueprint for Breast Cancer Predictive AI.

Modality Framework	Primary Input Data	Clinical Timing	Target End User	Intended Clinical Decision Support	Target Endpoint	Operational Unit of Analysis
Preoperative Radiomics/Deep Learning	Multiparametric/DCE-MRI volumes	Preoperative (Staging phase)	Breast Radiologists & Multidisciplinary Tumor Boards	Triage for neo-adjuvant planning and optimization of surgical axillary staging	Presence/Absence of Occult Preoperative LVI	Lesion-level or MRI Exam-level volume regions
Digital Pathology Channel Segmentation	H&E Core Needle Biopsy (CNB) or Resection WSIs (supplemented by D2-40/CD31 IHC)	Diagnostic Review (Pre- or Postoperative)	Surgical Pathologists	Screening for suspicious microemboli on H&E (noting that definitive route-specific differentiation requires auxiliary immunohistochemical validation or expert consensus).	Identification of composite LVI or suspicious tumor emboli against retraction artifacts on H&E (with explicit IHC confirmation required for true route-specific differentiation).	Patch-level/ROI-level pixels or micro-vessel counts
Pathology AI Auxiliary Screening	Axillary Sentinel/Non-Sentinel Lymph Node WSIs	Postoperative (Pathological Staging)	Surgical Pathologists	Rapid micro-metastasis screening to minimize observer fatigue and reduce review times	Nodal metastasis detection and burden stratification	Slide-level (Whole-Slide Image) or Nodal-level features
Multimodal/Clinicomics Pipelines	integrated Histology Slides + DCE-MRI + Biomarkers	Definitive Post-Surgical Evaluation	Medical Oncologists & Surgical Oncologists	Tailoring adjuvant chemotherapy duration and long-term surveillance intensity	5-Year Metastatic Relapse/Disease-Free Survival (DFS)	Patient-level composite risk score

**Table 3:** Structured Risk-of-Bias (QUADAS-AI) and Reporting Quality (CLAIM 2024) Appraisal Matrix.

Assessment Domain	Critical Appraisal Criteria & Framework Mapping	Identified Systemic Weaknesses in Reviewed Literature	Downstream Clinical Consequences & Mitigation Strategies
**Data Curation & Patient Selection** (*CLAIM/QUADAS-AI*)	Clear inclusion/exclusion criteria Handling of missing/corrupt data Multi-scanner, multi-institutional cohort representation	Severe over-reliance on single-center, retrospective data source streams. Missing data, scan artifact exclusions, and drop-out rates are rarely accounted for systematically.	Complete failure of technical reproducibility and cross-site portability testing. *Mitigation:* Implement rigorous open-science documentation alongside explicit operational explainability layers.
**Index Test & Model Description** (*CLAIM/QUADAS-AI*)	Detailed algorithmic architectures Disclosure of code, hyperparameters, and preprocessing states	Code repositories, data partitioning steps, and training random-seed states are infrequently published. Deep-learning features often act as black boxes lacking clear biological mapping.	Imperfect training labels reduce overall diagnostic truth and compromise calibration accuracy. *Mitigation:* Establish strict multi-pathologist consensus panels backed by confirmatory multiplex IHC
**Reference Standard Alignment** (*QUADAS-AI*)	Objective ground-truth definition Blinding of clinical annotators to algorithmic labels	Widespread variation between composite visual H&E evaluations, missing IHC validations (CD31/D2-40), and variable long-term survival window caps.	Introducing chronological confounding variables regarding tumor expansion or neo-adjuvant intervention changes. *Mitigation:* Enforce strict, standardized operational study windows within study inclusion criteria
**Flow and Timing** (*QUADAS-AI*)	Consistent time intervals between index tests and reference standards	Variations in the exact timing of pre-operative MRI scans relative to core needle biopsies or eventual surgical resections are rarely explicitly reported.	Severe, unrecognized algorithmic performance drop-offs when exposed to unseen clinical environments.
**Performance Evaluation & Portability** (*CLAIM*)	Independent, prospective, external geographic validation Evaluation of calibration, net clinical benefit, and subgroups	Evaluation is overwhelmingly constrained to internal split-sample cohorts or temporal validation. Reporting focuses almost completely on AUC, leaving calibration curves and net clinical benefit (DCA) underreported.	*Mitigation:* Implement rigorous open-science documentation alongside explicit operational explainability layers.

## Data Availability

No new data were acquired during the preparation of the paper.

## Abbreviations

The following abbreviations are used in this manuscript:

**AI**	Artificial Intelligence
**AUC**	Area Under the Receiver Operating Characteristic Curve
**CLAIM**	Checklist for Artificial Intelligence in Medical Imaging
**DCE-MRI**	Dynamic Contrast-Enhanced Magnetic Resonance Imaging
**DL**	Deep Learning
**FDA**	Food and Drug Administration
**HER2**	Human Epidermal Growth Factor Receptor 2
**IVDR**	In Vitro Diagnostic Medical Devices Regulation
**LBVI**	Lymph-Blood Vessel Invasion
**LightGBM**	Light Gradient Boosting Machine
**LVI**	Lymphovascular Invasion
**MLP**	Multilayer Perceptron
**MRI**	Magnetic Resonance Imaging
**MTL**	Multi-Task Learning
**QUADAS-AI**	Quality Assessment of Diagnostic Accuracy Studies–Artificial Intelligence
